# Research Quality of Clinical Trials Reported for Foods with Function Claims in Japan, 2023–2024: Evaluation Based on a Revised Tool to Assess Risk of Bias in Randomized Trials

**DOI:** 10.3390/nu16162744

**Published:** 2024-08-17

**Authors:** Hiroharu Kamioka, Jun Kitayuguchi, Hideki Origasa, Kiichiro Tsutani

**Affiliations:** 1 Faculty of Regional Environment Science, Tokyo University of Agriculture, 1-1-1 Sakuragaoka, Setagaya-ku 156-8502, Tokyo, Japan; 2 Physical Education and Medicine Research Center Unnan, 328 Uji, Unnan City 699-1105, Shimane, Japan; junk_907@yahoo.co.jp; 3 Institute of Statistical Mathematic, 10-3 Midori-cho, Tachikawa City 190-8562, Tokyo, Japan; origasahideki@gmail.com; 4 The Institute of Seizon and Life Sciences, 4-5-1 Ginza, Chuo-ku 104-0061, Tokyo, Japan; tsutanik@gmail.com

**Keywords:** healthy food, clinical trial, randomized controlled trial, risk of bias, functionality

## Abstract

Background: The Foods with Function Claim was introduced in Japan in April 2015 to make more products available that are labeled with health functions. A product’s functionality of function claims must be explained by the scientific evidence presented in clinical trials (CTs) or systematic reviews, but the quality of recent CTs is unclear. The purpose of this study was to evaluate the risk of bias (RoB) using “a revised tool to assess risk (RoB 2)” published in 2018 for notifications based on all recent CTs published on the Consumer Affairs Agency website. Methods: A total of 38 submitted papers based on CTs that were published on the Consumer Affairs Agency website during the period from 1 January 2023 to 30 June 2024 were eligible. The RoB 2 tool provides a framework for considering the risk of bias in the findings of any type of randomized trial. This tool with five domains was used to evaluate the quality of research methods. Results: Eligible CTs were assessed as “low risk” (11%, *n* = 4), “medium risk” (13%, *n* = 5), and “high risk” (76%, *n* = 29). A number of highly biased papers were published. Bias occurred in all five domains, especially “bias in selection of the reported result (Domain 5)”, which was the most serious (“high risk”; 75%). For elements correlated with RoB, there was no significant difference (*p* = 0.785) in the RoB 2 score between for-profit and academic research in the author’s affiliated organization. There was no significant difference (*p* = 0.498) in the RoB score between the published year categories of 2000–2019 and 2020–2024, and no significant difference (*p* = 0.643) in the RoB score between English and Japanese language publications. Conclusion: Overall, the quality of the latest CTs submitted after 2023 was very low, occurring in all five domains, and was most serious for “bias in selection of the reported result (Domain 5)”.

## 1. Introduction

In April 2015, Japan introduced Food with Function Claims (FFCs), a new type of food health claim to make it easier for consumers to obtain safe products with specific health functions clearly labeled [1]. Under the FFC system, manufacturers are permitted to sell their products by submitting to the Secretary-General of the Consumer Affairs Agency (CAA) claims that are expected to provide specific health functions and the scientific basis for these claims. Although the CAA does not evaluate the safety or functionality of notified products, applicants are required to complete all procedures necessary for notification [2]. All food products, including fresh produce, are subject to the FFC system. Prior to market entry (before at least 60 d), food business operators are required to submit information, such as food safety and functionality, to the system in place to collect information on adverse health effects and to the Secretary-General of CAA. The system is highly transparent, and all information submitted, including withdrawals and amendments, is made publicly available on the CAA website. In order to prove the functionality of a product, its scientific evidence needs to be described with positive results from either clinical trials (CTs), such as randomized controlled trials (RCTs), or systematic reviews (SRs). In fact, since the system’s launch, 8461 cases have been reported as of 30 June 2024, of which 326 cases (4%) used CTs and 8115 cases (96%) used SRs [3].

However, one of the weaknesses of the FFC system is the adequacy and rigor of the scientific evidence. Given this concern, the CAA undertook national verification projects [4,5], and some scholar groups evaluated research methodologies [6,7,8,9,10] and reporting methods [11].

Regarding SRs, in 2016, the CAA established a working group of experts (SR methodologists) to verify whether submitted SRs are clearly described in accordance with the first edition of the PRISMA checklist [12]. As a result, many SRs submitted had multiple items omitted or inadequate explanations due to an insufficient understanding of the required content [4]. Academic researchers [6] conducted a quality evaluation of SRs using the first edition of ‘assessment of multiple systematic reviews’ (AMSTARs) [13] reports [10,12]. They found very poor descriptions and/or the implementation of study selection, data extraction, search strategies, evaluation methodology for risk of bias (RoB), assessment of publication bias, and the formulation of conclusions based on the methodological rigor and scientific quality of the included CTs. Furthermore, a recent study [7] reported on the methodological quality of SRs evaluated by the AMSTAR 2 checklist [14]. In that study, 40 SRs were randomly extracted based on eligibility criteria and recruitment procedures. Overall confidence for the SRs was rated as “High” (N = 0, 0%), “Moderate” (N = 0, 0%), “Low” (N = 2, 5%), or “Critically low” (N = 38, 95%). Registering the review protocol and using comprehensive search strategies are particularly common deficiencies. Additionally, RoB was insufficiently considered.

The CAA investigated 50 CTs reported in 2017 and found that many had inadequate protocols, unclear methods of RoB assessment, and conflicts of interest issues [5]. One study [8] examined how accurately 33 RCTs described the 29 required items on the CONSORT 2010 checklist [15] and concluded that an average of 13.8 out of 29 items (47.6%) had sufficient descriptions. A study in 2021 identified many studies in which it was unclear whether the protocols were followed in the CTs that had been submitted and also found issues with selective reporting and the intentional concealment of the intervention content (test foods) [9]. A subsequent 2022 study by these same researchers reported that randomization, deviations from intended interventions, the measurement of outcomes, and selective reporting (in particular, RoB, including the lack of intention-to-treat [ITT] analysis, unknown compliance and multiple outcome tests) seriously damaged the study quality [10]. A recent meta-epidemiological study evaluated the quality of RCTs facilitated by prominent contract research organizations in Japan and examined the quality of these representations used to convey their results to consumers [11]. It was reported that approximately 72% of the RCT publications exhibited a high RoB due to selective outcome reporting, and ‘‘spin’’ appeared in 73% of press releases/advertisements due to selective outcome reporting. Thus, the reported CTs had many research methodological problems despite being published in academic journals. Research that has a high RoB is problematic because it is scientifically unsound and can mislead readers.

There have been previous studies that evaluated the quality of notified CTs using the “Revised Cochrane risk-of-bias tool for randomized trials (RoB 2)” [16]. However, these were not exhaustive surveys, included many CTs published in older years, and did not investigate the relationship with other factors.

Therefore, the present study aimed to evaluate the RoB using RoB 2 for notifications based on all recent CTs published on the CAA website from 1 January 2023 to 30 June 2024 and to clarify the characteristics of the CTs themselves as well as other factors.

## 2. Methods

### 2.1. Eligibility and Exclusion Criteria (Target Article)

All submitted papers based on CTs published on the CAA website during the period from 1 January 2023 to 30 June 2024* were eligible. We set this period because we believe that the more recent CTs are of higher quality than earlier ones. Papers based on SRs and observational studies were excluded. Intervention studies (single arm) without a comparison group were excluded as a research design. If there were overlaps, such as multiple notifications using the same paper as proof of functionality, only the first paper was accepted. In SRs, authors must verify items that are not mentioned in a paper. However, in this study, we were focusing only on understanding whether there was bias in a target paper, so if the essential information was not stated in the paper, we did not confirm the contents with the authors.

*The health hazards detected in March 2024 that were caused by supplements manufactured and sold by KOBAYASHI Pharmaceutical Co., Ltd. have become a major social issue [17]. As a result, our study period could be extended if the number of samples were extremely small due to delays in notifications to the CAA. Therefore, in the middle of our ongoing study, we revised the protocol to include a start time of 1 January 2023 instead of 1 January 2024.

### 2.2. Data Extraction Source

We downloaded target articles from the CAA website.

### 2.3. Data Item and Evaluation of Methodological Quality (RoB Score)

The RoB 2 used to evaluate the quality of research methods included (i) bias resulting from the randomization process, (ii) bias due to deviations from the intended intervention, (iii) bias due to missing outcomes, (iv) bias in measurement/evaluation, and (v) bias in the selection of reported results (for details of the method, refer to the original literature [16]). The RoB 2 tool provides a framework for considering RoB in the findings of any type of randomized trial. The evaluation procedure was carried out in accordance with the RoB 2 manual. The crossover trial was evaluated using the RoB 2 preliminary tool version [18].

Each domain was evaluated in three stages: “low risk”, “medium risk (somewhat suspicious)”, and “high risk”. The criteria were implemented in accordance with the original published guidance [16,18]. In accordance with the items covered in previous studies [9,10], for each targeted study, we also searched the affiliation characteristics of the first author (for-profit researcher or academic researcher), the year in which the paper was published, the language of the paper (English or Japanese), and the impact factor (IF) in 2022. The IF was assessed according to the Clarivate Analytics’ gate (https://jcr.clarivate.com/) (Accessed on 1 June 2024). For journals that did not have an IF, we quantified the IF as 0.

### 2.4. Summary Scale

To compute the overall RoB 2 score and each domain score, low risk was quantified as 1, medium risk as 2, and high risk as 3.

### 2.5. Statistical Analysis

The RoB 2 score (1–3) and each domain score (1–3) were used as dependent variables. The author’s affiliation (for-profit researcher or academic researcher), year of publication (before 2019 or after 2020), language characteristics (English or Japanese), and IF were used as explanatory variables. The three items mentioned above were tested using Fisher’s exact test and the Kruskal–Wallis test. All statistical analyses were performed with SPSS Statistics 25.0 (IBM Corporation, Armonk, NY, USA). *p*-values less than 0.05 were considered statistically significant.

### 2.6. Protocol Registration

The present study’s methodology (protocol) was established on 3 April 2024. The study was registered as UMIN 000,054,051 by the University Hospital Medical Information Network Clinical Trials Registry (UMIN-CTR)* in Japan (refer: https://center6.umin.ac.jp/cgi-open-bin/ctr/ctr_view.cgi?recptno=R000061712) (Accessed on 1 June 2024). However, UMIN-CTR could not register the contents of all protocols in the input settings, so the complete protocol was stored in an online cloud, which can be viewed from this link: https://1drv.ms/b/s!AoQmpnIHE3YUhNMaSGl3ydWdcwiwuA?e=gdFDVb (Accessed on 1 June 2024).

*UMIN-CTR is the largest CTR in Japan and joined the WHO registry network in October 2008.

## 3. Results

### 3.1. Study Selection and Characteristics

**Figure 1:**
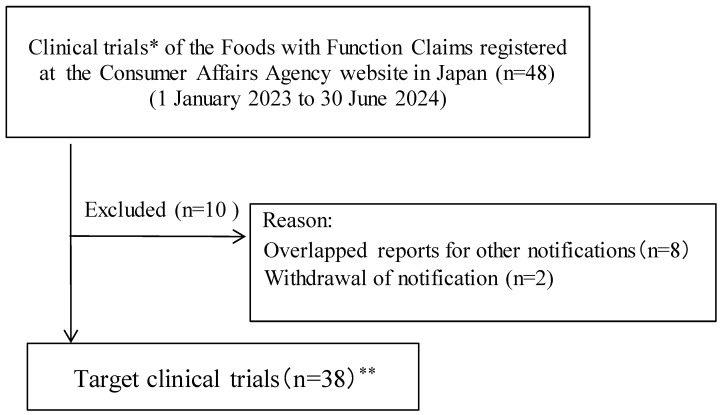
Flowchart of the trial process and study implementation. * Number of notifications for Food with Function Claims (*n* = 42). ** Additional information can be found at the following URL: https://1drv.ms/b/s!AoQmpnIHE3YUhNsckPJfab7b9FJyeA?e=2WB4ff (Accessed on 1 July 2024).

Preliminary research identified 48 applicable publications, of which 38 met the eligibility criteria before final confirmation (Figure 1 and Supplementary Table S1). Eligible articles were published in 14 journals, and most (68%) were published in 2020–2024 (Table 1). The languages of eligible publications were English (55%) and Japanese (45%). According to the affiliation classification of the first author, for-profit research comprised 84%, and academic research comprised 16%. Seventy-three percent of journals had no IF.

### 3.2. Feature of RoB 2 Score and Each Domain Score

**Figure 2:**
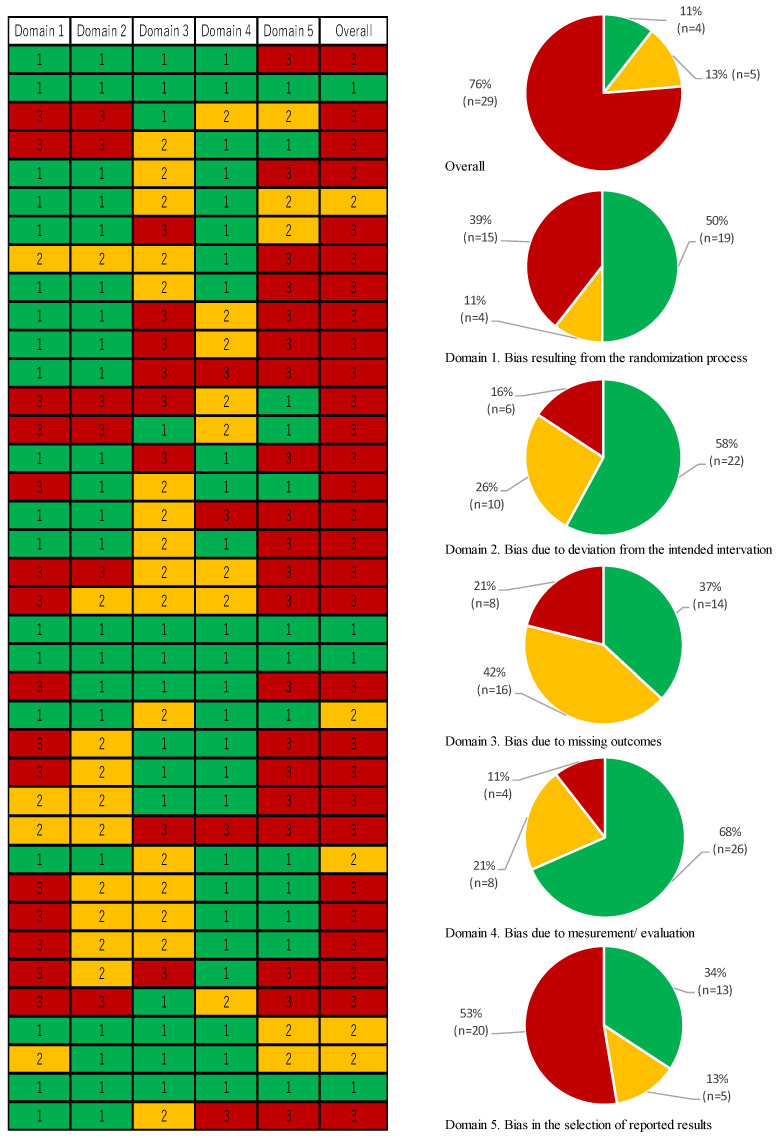
Feature of RoB 2 score and each domain score. Note: All heat maps show “low risk” in green, “medium risk” in yellow, and “high risk” in red.

Figure 2 shows that the overall RoB 2 assessment on target articles was “low risk” (11%, *n* = 4), “medium risk” (13%, *n* = 5), and “high risk” (76%, *n* = 29).

Regarding each domain score, bias in the articles that resulted from the randomization process was “low risk” (50%, *n* = 19), “medium risk” (11%, *n* = 4), and “high risk” (39%, n = 15). Bias due to deviation from the intended intervention was “low risk” (58%, *n* = 22), “medium risk” (26%, *n* = 10), and “high risk” (16%, *n* = 6). Bias due to missing outcomes was “low risk” (37%, *n* = 14), “medium risk” (42%, *n* = 16), and “high risk” (21%, *n* = 8). Bias in measurement/evaluation was “low risk” (68%, *n* = 26), “medium risk” (21%, *n* = 8), and “high risk” (11%, *n* = 4). And bias in the selection of reported results was “low risk” (34%, *n* = 13), “medium risk” (13%, *n* = 5), and “high risk” (53%, *n* = 20).

### 3.3. Elements Correlated with RoB

#### 3.3.1. RoB 2 Score

There was no significant difference (*p* = 0.785) between for-profit and academic research in the author’s use of the RoB 2 score (Table 2). There was no significant difference (*p* = 0.498) in the RoB score between the published year categories of 2000–2019 and 2020–2024, and no significant difference (*p* = 0.643) in the RoB score between English and Japanese language publications. Regarding the RoB score and IF, the Kruskal–Wallis test showed no significant difference (*p* = 0.312).

#### 3.3.2. Each Domain Score

Concerning bias resulting from the randomization process, there was a significant difference (*p* = 0.018) in the RoB score between for-profit and academic research in the author’s organization (Table 3). Also, there was a significant difference (*p* = 0.006) in the RoB score between the published year categories of 2000–2019 and 2020–2024 and a significant difference (*p* = 0.031) in the RoB score between English and Japanese language publications. There was no significant difference in the RoB score for IF (*p* = 0.989).

Regarding bias due to deviation from the intended intervention, there was a significant difference (*p* = 0.002) in the RoB score between for-profit and academic research in the author’s organization. In other items, there were no significant differences.

There was no significant difference in all items for bias due to missing outcomes, bias in measurement/evaluation, and bias in the selection of reported results.

All heat maps show “low risk” in green, “medium risk” in yellow, and “high risk” in red.

## 4. Discussion

This was the first study to evaluate CTs reported as scientific evidence of efficacy in the FFC system, using the RoB 2 tool, and identify associated factors. Unfortunately, the CTs tended to have high RoB. As in other healthcare fields, nutritional SRs are best conducted to synthesize data from CTs. Therefore, SRs that collect low-quality CTs cannot draw valid conclusions about food functionality. Such conclusions lead consumers to make poor decisions when purchasing products that claim to have certain functionalities.

### 4.1. Features of RoB on CTs

Bias is defined as a systematic error in study results and is caused by incorrect research methodology [19]. In observational studies, such as cross-sectional, cohort, and case–control studies, it is well-known that confounding factors are biases that have a greater impact on outcomes. In this study, bias occurred in all five domains and was most serious for “bias in selection of the reported result (Domain 5)”. Due to unclear descriptions of outcomes in the protocols, there were many inconsistencies in the outcomes reported in the articles. This finding is consistent with previous studies evaluating protocol compliance [9]. Also, multiple outcome measures (e.g., scales, definitions, and time points) were utilized; thus, the multiplicity of outcome items was the most serious problem. If this bias was high, the appropriateness of the clinical trial itself could be seriously questioned. In particular, in addition to selective reporting, a new problem identified was that the content of the intervention (test food) was intentionally concealed. This problem was also previously reported in a review of funding for pharmaceutical industry studies [20]. In the guidelines, it may be necessary to make it compulsory not to accept papers that do not have a detailed description of the protocol.

“Bias arising from the randomization process (Domain 1)” is a very common form of miss-reporting. In many papers, the specific randomization method was unclear, with “allocation sequence concealed”. A study evaluating the RoB of 10,103 trials reported frequent random sequence generation that did not follow the instructions in the Cochrane Handbook [21]. A related study suggested that the blinding of participants and personnel (performance bias) was also frequently not in line with the Handbook recommendations [22]. These findings highlight the RoB domain as a potential pitfall in various kinds of CTs, and researchers should diligently avoid it, beginning at the planning stage.

In addition, “bias due to missing outcome data (Domain 3)” was high. Because CTs of health foods generally have a relatively short intervention period (most often 8–12 weeks [8]), it is necessary to analyze the ITT population or the full-set population.

### 4.2. Elements Correlated with RoB

The sub-research questions assessed in this study were as follows: “Is RoB correlated with author characteristics (for-profit corporate authors and academic authors), the year in which the paper was published, the relationship between English and other languages, and the IF”. Overall, RoB 2 scores were not associated with the author affiliation, year of publication, language, or IF. However, academic researchers tended to be significantly more biased in two domains than for-profit researchers. The reason for this may be related to the fact that the year of a publication by an academic researcher is older than that of a for-profit researcher. In fact, in bias resulting from the randomization process, 75% of papers published in 2000–2019 were high risk compared with 23% of papers published in 2020–2024. This suggests that there are many defects in partial domains in older papers.

### 4.3. Impact on SRs

In the FFC system, approximately 95% of notifications are submitted using SRs [6,7]. A recent study demonstrated that the quality of SRs is extremely poor [7]. In fact, CTs on functional ingredients have been included as target papers in SRs. SRs must have little RoB in the reviewed CTs, so our findings bring into question the reliability and quality of SRs in the FFC system. When determining the credibility of study results by meta-analysis, it is very important to know whether only low RoB CTs are included or high RoB CTs are excluded. For example, a previous study that evaluated 59 SRs reported that only 50% of the SRs performed sensitivity analyses for low RoB CTs [23].

The CAA has issued updated guidelines [2] requiring all newly filed SRs (including updated SRs) to comply with PRISMA 2020 [24]. When reporting an SR based on PRISMA 2020, the assessment of the certainty of the evidence is paramount for the final conclusion. Consequently, the Grading of Recommendations Assessment, Development, and Evaluation (GRADE) theory is often followed [25]. The GRADE method has already been adopted in the nutrition- and food-related fields [26,27,28]. A methodological review of nutrition SRs [29] reported that among 800 SRs, 55 used the GRADE method; certainty of evidence has been downgraded mostly for the RoB (37.8%) in the SRs of RCTs. In the FFC-SR system, proper evaluation of the RoB of each CT is an absolute requirement for the implementation of an SR. Conducting RoB assessments to validate SR findings imposes high demands on reviewers’ expertise as well as on resources such as time and cost. However, it is doubtful whether the appropriate evaluation method is rigorously implemented for SRs [6,7]. The results of the present study reveal that, in addition to the low quality of the individual CTs, there are methodological problems with the SR research conducted by extracting the CTs, which is a fundamental problem for the FFC system.

### 4.4. Future Research Challenges to Improve the Quality of CT on the FFC

In the planning stage for conducting a CT, researchers need to carefully review some type of RoB 2 checklist [16,18], as well as the current reporting guidelines (i.e., SPIRIT 2013 [30], CONSORT 2010 statement [15,31], and CONSORT 2010 statement: crossover extension [32]) and take steps to avoid bias. The SPIRIT 2024 and the CONSORT 2024 guidelines are expected to be published soon for CT protocols and reporting methods [33], so any new CTs will need to comply.

**Figure 3:**
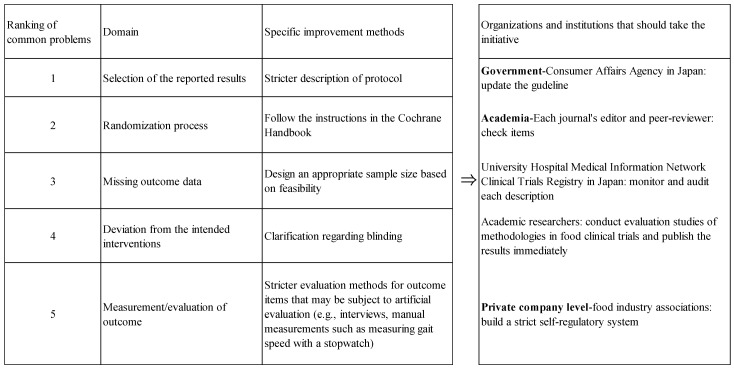
Challenges to strengthen the research quality of food-related clinical trials.

We assume that in order to conduct a high-quality CT on food in the future, it will be necessary not only for the researchers themselves to improve their processes but also for a tripartite effort by the government, academic organizations/researchers, and the food industry will be needed (Figure 3). The frequency order of these domains that were particularly inaccurate or inappropriate was “selection of the reported results”, “missing outcome data”, and “randomization process”, and a common problem was a lack of rigor in the items that needed to be reported. The CCA may need to clearly state in its guidelines that studies with a high RoB above a certain level will not be able to apply for the FFC system. It may be an effective measure for nutrition- and food-related journals to incorporate RoB into their editorial and review policies. Of course, journal editors should reject low-quality research to avoid future misunderstandings in the field. At this time, UMIN-CTR is not reviewing the contents of protocols or requesting corrections if there are any deficiencies. Identifying flaws at the protocol stage may help to avoid some of the problems of low-quality papers being published. We consider that academic researchers need to be more critical in peer-reviewing research papers, and they should promote an evaluation of clinical trial methodologies for food. Food industry associations should place importance on evidence that contradicts the sale of large numbers of functional foods and comply with high standards of research ethics. One way they could undertake this would be to institute self-regulation in order to reduce RoB.

### 4.5. Challenges in Building a Bridge with End Users (Consumers)

In the FFC system, if a positive result is obtained for a CT’s functionality, it will be accepted as a notification of functionality. It is also true that the contents of CTs and their interpretations are difficult for the average consumer to understand. Since CTs often have low-quality notifications, it is necessary to correctly communicate this information to consumers in order to make appropriate purchasing decisions. Consumers probably have no idea what RoB means, so they will accept the results presented in an academic paper. One previous study pointed out that academic researchers, responsible authorities, and relevant government agencies need to work together to properly convey this information to consumers so that they can make appropriate purchasing decisions [34].

To achieve this aim, it may be necessary to (i) present the content of the CT in plain language; (ii) provide a method for academic researchers or responsible authorities to include easy-to-understand comments on the paper’s RoB 2 evaluation; and (iii) develop human resources and create a system to further educate consumers on how to scientifically interpret these evaluations.

## 5. Limitations

There were several limitations to the present study. First, we only focused on CTs based on the notification to the FFC in Japan (a single country), so our findings may not necessarily be generalized to all CTs of healthy foods. In fact, about half of the articles in our study were written in Japanese. Second, since it targets the latest CTs, the 38 articles included in our study were a relatively medium sample size. Third, although there could be many other potential elements related to RoB, we only assessed the following four aspects: first author characteristics, published year, languages, and the IF. In other words, in the case of authors of companies, and where the published paper was old, a Japanese paper was not written in English, the IF was low, or there was no IF, it was assumed that the RoB was high. Fourth, we could not describe the results of a quality assessment based on the food business operator’s real name (i.e., identified for a product) because of the potential risk of civil suits and other serious issues. However, target articles were listed as supplementary data and can be found online at: https://1drv.ms/b/s!AoQmpnIHE3YUhNsckPJfab7b9FJyeA?e=2WB4ff (Accessed on 1 July 2024).

Finally, a meta-epidemiological study suggested that many SRs did not adhere to the RoB 2 guidance because they applied the tool at the study level rather than at the outcome measure level [35]. However, our study adapted the RoB 2 tool to evaluate the quality of CTs. Our RoB evaluation was performed by only one author (i.e., HK) who was fully experienced in the assessment of CT quality, which may have introduced some errors. A recent study using RoB 2 reported that the tool and its guidance are useful but resource-intensive and challenging to implement [36]. In addition, this study notes that despite the extensive guidance, it was difficult to implement aspects of most domains. In other words, this suggests that it is difficult to make a judgment without more detailed explanations for each domain. A previous study estimated the time taken to apply the RoB 2 tool, finding that it was demanding with problematic reliability, and therefore, recommended the development of operational criteria specific to the review to improve implementation [37]. A recent study summarized findings from other studies that evaluated the design and usability of RoB tools such as the Prediction model Risk Of Bias ASessment Tool (PROBAST), RoB2, Risk Of Bias In Non-randomized Studies of Interventions (ROBINS-I), the Quality Assessment of Diagnostic Accuracy Studies-2 (QUADAS-2), and others [38]. It reported that these evaluation tools have methodological limitations restricting the generalizability of their findings. As such, there are common challenges and limitations in any tools when evaluating RoB.

## 6. Conclusions

The quality of the most recent CTs submitted after 2023 under the Japanese FFC system is very low. In particular, there are three common biases in most CTs that have been reported as the scientific basis of efficacy in the FFC system: selection of the reported result, randomization process, and missing outcome data.

In the planning stages of their study’s conduct, researchers in the nutrition field need to carefully review some types of RoB 2 checklists as well as reporting guidelines and should take all necessary steps to avoid bias. Since the evaluation items of RoB 2 are listed, their use as a checklist can be considered a concrete way to create a protocol.

## Figures and Tables

**Table 1:** Published journals’ characteristics.

Journal Name	N
Japanese Pharmacological and Therapeutics/薬理と治療	20 (53%)
Functional Foods in Health and Disease	4 (11%)
Medical Consultation and New Remedies/診療と新薬	3 (8%)
	Common to all of the following journals: 1 (3%)
Biological and Pharmaceutical Bulletin	
Clinical, Cosmetic and Investigational Dermatology	
Food Science & Nutrition Research	
Frontiers in Nutrition	
Immunology, Endocrine & Metabolic Agents in Medicinal Chemistry	
Integrative Molecular Medicine	
Journal of Clinical Biochemistry and Nutrition	
Journal of Functional Foods	
Journal of Fungi	
Nutrients	
Progress in Medicine	
	
Published year	
2000–2019	12 (32%)
2020–2024	26 (68%)
Language	
English	21 (55%)
Japanese	17 (45%)
Category of first author’s organization	
For-profit	32 (84%)
Academic	6 (16%)
Journal’s impact factor in 2022	
None (0)	28 (73%)
1.999>	3 (8%)
2.000–3.999	3 (8%)
>4.000	4 (11%)
Value: *n* (%)	

**Table 2:** Elements correlated with RoB 2 score.

		Low Risk	Medium Risk	High Risk	*p*-Value *
Total		11% (N = 4)	13% (N = 5)	76% (N = 29)	*-*
Author’s affiliation					
	For-profit	12.5% (N = 4)	15.6% (N = 5)	71.9% (N = 23)	0.785
	Academic	0.0% (N = 0)	0.0% (N = 0)	100.0% (N = 6)	
Year of publication					
	-2019	0.0% (N = 0)	16.7% (N = 2)	83.3% (N = 10)	0.498
	2020–2024	15.4% (N = 4)	11.5% (N = 3)	73.1% (N = 19)	
Language					
	English	14.3% (N = 3)	9.5% (N = 2)	76.2% (N = 16)	0.643
	Japanese	5.9% (N = 1)	17.6% (N = 3)	76.5% (N = 13)	
Impact factor **		1.0 (0.3–1.0) (N = 4)	0.0 (0.0–0.5) (N = 5)	0.0 (0.0–1.0) (N = 29)	0.312

* Fisher’s exact test and Kruskal–Wallis test. ** Median (interquartile range).

**Table 3:** Elements correlated with each bias domain.

			Low Risk	Medium Risk	High Risk	*p*-Value *
Domain 1: Bias resulting from the randomization process						
Author’s affiliation		For-profit	59.4% (N = 19)	9.4% (N = 3)	31.3% (N = 10)	0.018
		Academic	0.0% (N = 0)	16.7% (N = 1)	83.3% (N = 5)	
						
Year of publication		-2019	16.7% (N = 2)	8.3% (N = 1)	75.0% (N = 9)	0.006
		2020–2024	65.4% (N = 17)	11.5% (N = 3)	23.1% (N = 6)	
						
Language		English	38.1% (N = 8)	4.8% (N = 1)	57.1% (N = 12)	0.031
		Japanese	64.7% (N = 11)	17.6% (N = 3)	17.6% (N = 3)	
						
Impact factor **			0.0 (0.0–1.0) (N = 19)	0.0 (0.0–4.4) (N = 4)	0.0 (0.0–2.0) (N = 15)	0.989
						
Domain 2: Bias due to deviation from the intended intervention						
Author’s affiliation		For-profit	68.8% (N = 22)	21.9% (N = 7)	9.4% (N = 3)	0.002
		Academic	0.0% (N = 0)	50.0% (N = 3)	50.0% (N = 3)	
						
Year of publication		-2019	33.3% (N = 4)	41.7% (N = 5)	25.0% (N = 3)	0.100
		2020–2024	69.2% (N = 18)	19.2% (N = 5)	11.5% (N = 3)	
						
Language		English	42.9% (N = 9)	38.1% (N = 8)	19.0% (N = 4)	0.084
		Japanese	76.5% (N = 13)	11.8% (N = 2)	11.8% (N = 2)	
						
Impact factor **			0.0 (0.0–1.0) (N = 22)	0.0 (0.0–1.3) (N = 10)	1.0 (0.0–4.9) (N = 6)	0.349
Domain 3: Bias due to missing outcomes						
Author’s affiliation		For-profit	37.5% (N = 12)	37.5% (N = 12)	25.0% (N = 8)	0.388
		Academic	33.3% (N = 2)	66.7% (N = 4)	0.0% (N = 0)	
						
Year of publication		-2019	41.7% (N = 5)	41.7% (N = 5)	16.7% (N = 2)	1.000
		2020–2024	34.6% (N = 9)	42.3% (N = 11)	23.1% (N = 6)	
						
Language		English	33.3% (N = 7)	52.4% (N = 11)	14.3% (N = 3)	0.311
		Japanese	41.2% (N = 7)	29.4% (N = 5)	29.4% (N = 5)	
						
Impact factor **			0.0 (0.0–1.0) (N = 14)	0.0 (0.0–2.1) (N = 16)	0.0 (0.0–0.0) (N = 8)	0.530
						
Domain 4: Bias in measurement/evaluation						
Author’s affiliation		For-profit	71.9% (N = 23)	15.6% (N = 5)	12.5% (N = 4)	0.200
		Academic	50.0% (N = 3)	50.0% (N = 3)	0.0% (N = 0)	
						
Year of publication		-2019	75.0% (N = 9)	16.7% (N = 2)	8.3% (N = 1)	1.000
		2020–2024	65.4% (N = 17)	23.1% (N = 6)	11.5% (N = 3)	
						
Language		English	66.7% (N = 14)	23.8% (N = 5)	9.5% (N = 2)	1.000
		Japanese	70.6% (N = 12)	17.6% (N = 3)	11.8% (N = 2)	
						
Impact factor **			0.0 (0.0–0.3) (N = 26)	1.0 (0.0–4.9) (N = 8)	0.0 (0.0–1.8) (N = 4)	0.198
						
Domain 5: Bias in the selection of reported results						
Author’s affiliation		For-profit	37.5% (N = 12)	12.5% (N = 4)	50.0% (N = 16)	0.584
		Academic	16.7% (N = 1)	16.7% (N = 1)	66.7% (N = 4)	
						
Year of publication		-2019	41.7% (N = 5)	25.0% (N = 3)	33.3% (N = 4)	0.164
		2020–2024	30.8% (N = 8)	7.7% (N = 2)	61.5% (N = 16)	
						
Language		English	47.6% (N = 10)	9.5% (N = 2)	42.9% (N = 9)	0.163
		Japanese	17.6% (N = 3)	17.6% (N = 3)	64.7% (N = 11)	
						
Impact factor **			0.0 (0.0–1.0) (N = 13)	0.0 (0.0–1.0) (N = 5)	0.0 (0.0–1.7) (N = 20)	0.836

* Fisher’s exact test and Kruskal–Wallis test. ** Median (interquartile range).

## Data Availability

Data Availability Statements are available in section “MDPI Research Data Policies” at https://1drv.ms/b/s!AoQmpnIHE3YUhNsckPJfab7b9FJyeA?e=2WB4ff (Accessed on 1 July 2024).

## Supplementary Materials

The following supporting information can be downloaded at https://1drv.ms/b/s!AoQmpnIHE3YUhNsckPJfab7b9FJyeA?e=2WB4ff (Accessed on 1 July 2024). Table S1: Characteristics of clinical trials of Foods with Function Claims.

## Abbreviations

CAA: Consumer Affairs Agency; CT: clinical trial; CTR: clinical trial registration; FFC: Foods with Function Claims; IF: impact factor; PICOS: Participant, Intervention, Comparison, Outcome, and Study design; PROBAST: Prediction model Risk of Bias ASessment Tool; RCT: randomized controlled trial; RoB: risk of bias; RoB 2: Revised Tool to Assess Risk of Bas in Randomized Trials; ROBINS-I: Risk Of Bias In Non-randomized Studies of Interventions; QUADAS-2: Quality Assessment of Diagnostic Accuracy Studies-2; SR: systematic review; UMIN-CTR: University Hospital Medical Information Network Clinical Trials Registry; and WHO: World Health Organization.

## References

[1] Consumer Affairs Agency. Government of Japan (2015). Introduction. https://www.caa.go.jp/policies/policy/food_labeling/information/pamphlets/pdf/151224_2.pdf.

[2] Consumer Affairs Agency. Government of Japan Guideline (Updated April 2023). https://www.caa.go.jp/policies/policy/food_labeling/foods_with_function_claims/assets/foods_with_function_claims_210322_0002.pdf.

[3] Consumer Affairs Agency. Government of Japan Notification Information Search Site. https://www.fld.caa.go.jp/caaks/cssc01/.

[4] Consumer Affairs Agency. Government of Japan (2016). Verification of Scientific Evidence on Effectiveness of the System of “Foods with Function Claim”: Assessment of the Submitted Systematic Literature Reviews (Digest Edition). https://www.caa.go.jp/policies/policy/food_labeling/foods_with_function_claims/pdf/about_food_with_function_report_180416_0001.pdf.

[5] Consumer Affairs Agency. Government of Japan Verification of Scientific Evidence on “Foods with Function Claims”: Assessment of the Submitted Clinical Trials. https://www.caa.go.jp/policies/policy/food_labeling/foods_with_function_claims/pdf/foods_index_23_171025_0001.pdf.

[6] Kamioka H., Tsutani K., Origasa H., Yoshizaki T., Kitayuguchi J., Shimada M., Wada Y., Takano-Ohmuro H. (2019). Quality of systematic reviews of the Foods with Function Claims in Japan: Comparative before- and after-evaluation of verification reports by the Consumer Affairs Agency. Nutrients.

[7] Kamioka H., Origasa H., Tsutani K., Kitayuguchi J., Yoshizaki T., Shimada M., Wada Y., Takano-Ohmuro H. (2023). A cross-sectional study based on forty systematic reviews of Foods with Function Claims (FFC) in Japan: Quality assessment using AMSTAR 2. Nutrients.

[8] Tanemura N., Hamadate N., Urushibara H. (2018). Evaluation of randomized controlled trials of foods with functional claims re-quest: The learning outcomes from studies in Japan. J. Funct. Foods.

[9] Kamioka H., Origasa H., Kitayuguchi J., Tsutani K. (2022). Compliance of clinical trial protocols for Foods with Function Claims (FFC) in Japan: Consistency between clinical trial registrations and published reports. Nutrients.

[10] Kamioka H., Origasa H., Kitayuguchi J., Yoshizaki T., Shimada M., Wada Y., Takano-Ohmuro H., Tsutani K. (2022). Risk of bias in clinical trials reported for Foods with Functional Claims in Japan: A cross-sectional study on research quality. J. Clin. Trials.

[11] Someko H., Yamamoto N., Ito T., Suzuki T., Tsuge T., Yabuzaki H., Dohi E., Kataoka Y. (2024). Misleading presentations in functional food trials led by contract research organizations were frequently observed in Japan: Meta-epidemiological study. J. Clin. Epidemiol..

[12] Liberati A., Altman D.G., Tetzlaff J., Mulrow C., Gotzsche P.C., Ioannidis J.P., Clarke M., Devereaux P.J., Kleijnen J., Moher D. (2009). The PRISMA statement for reporting systematic reviews and meta-analyses of studies that evaluate health care interventions: Explanation and elaboration. Ann. Intern. Med..

[13] Shea B.J., Grimshaw J.M., A Wells G., Boers M., Andersson N., Hamel C., Porter A.C., Tugwell P., Moher D., Bouter L.M. (2007). Development of AMSTAR: A measurement tool to assess the methodological quality of systematic reviews. BMC Med. Res. Methodol..

[14] Shea B.J., Reeves B.C., Wells G., Thuku M., Hamel C., Moran J., Moher D., Tugwell V., Kristjansson E., Henry D.A. (2017). AMSTAR 2: A critical appraisal tool for systematic reviews that include randomized or non-randomized studies of healthcare interventions, or both. BMJ.

[15] Schulz K.F., Altman D.G., Moher D., CONSORT Group (2010). CONSORT 2010 statement: Updated guidelines for reporting parallel group randomised trials. PLoS Med..

[16] Higgins J.P.T., Savović J., Page M.J., Sterne J.A.C., on behalf of the RoB 2 Development Group Revised Cochrane Risk-of-Bias Tool for Randomized Trials (RoB 2). Published on 22 August 2019. https://drive.google.com/file/d/19R9savfPdCHC8XLz2iiMvL_71lPJERWK/view?pli=1.

[17] Hashimoto T., Ozaki A., Hakariya H., Takahashi K., Tanimoto T. (2024). The Beni-Koji scandal and Japan’s unique health food system. Lancet.

[18] Higgins J.P.T., Li T., Sterne J., the RoB 2 Working Group on Crossover Trials Revised Cochrane Risk of Bias Tool for Randomized Trials (RoB 2): Additional Considerations for Crossover Trials (Preliminary Tool Version, 18 March 2021). https://www.riskofbias.info/welcome/rob-2-0-tool/rob-2-for-crossover-trials.

[19] Higgins J.P., Altman D.G., Gøtzsche P.C., Jüni P., Moher D., Oxman A.D., Savovic J., Schulz K.F., Weeks L., Sterne J.A.C. (2011). Cochrane Bias Methods Group; Cochrane Statistical Methods Group. The Cochrane Collaboration’s tool for assessing risk of bias in randomised trials. BMJ.

[20] Melander H., Ahlqvist-Rastad J., Meijer G., Beermann B. (2003). Evidence b(i)ased medicine-selective reporting from studies sponsored by pharmaceutical industry: Review of studies in new drug applications. BMJ.

[21] Barcot O., Boric M., Pericic T.A., Cavar M., Dosenovic S., Vuka I., Puljak L. (2019). Risk of bias judgments for random sequence generation in Cochrane systematic reviews were frequently not in line with Cochrane Handbook. BMC Med. Res. Methodol..

[22] Barcot O., Boric M., Dosenovic S., Pericic T.P., Cavar M., Puljak L. (2019). Risk of bias assessments for blinding of participants and personnel in Cochrane Reviews were frequently inadequate. J. Clin. Epidemiol..

[23] Katikireddi S.V., Egan M., Petticrew M. (2015). How do systematic reviews incorporate risk of bias assessments into the synthesis of evidence? a methodological study. J. Epidemiol. Community Health.

[24] Page M.J., McKenzie J.E., Bossuyt P.M., Boutron I., Hoffmann T.C., Mulrow C.D., Shamseer L., Tetzlaff J.M., Aki E.A., Brennan S.E. (2021). The PRISMA 2020 statement: An updated guideline for reporting systematic reviews. BMJ.

[25] Hultcrantz M., Rind D., Akl E.A., Treweek S., Mustafa R.A., Iorio A., Alper B.S., Meerpohl J.J., Murad M.H., Ansari M.T. (2017). The GRADE Working Group clarifies the construct of certainty of evidence. J. Clin. Epidemiol..

[26] Santesso N., Akl E.A., Bianchi M., Mente A., Mustafa R., Heels-Ansdell D., Schünemann H.J. (2012). Effects of higher-versus lower-protein diets on health outcomes: A systematic review and meta-analysis. Eur. J. Clin. Nutr..

[27] De Souza R.J., Mente A., Maroleanu A., Cozma A.I., Ha V., Kishibe T. (2015). Intake of saturated and trans unsaturated fatty acids and risk of all-cause mortality, cardiovascular disease, and type 2 diabetes: Systematic review and meta-analysis of observational studies. BMJ.

[28] Reynolds A., Mann J., Cummings J., Winter N., Mete E., Te Morenga L. (2019). Carbohydrate quality and human health: A series of systematic reviews and meta-analyses. Lancet.

[29] Werner S.S., Binder N., Toews I., Schünemann H.J., Meerpohl J.J., Schwingshackl L. (2021). Use of the GRADE approach for rating the certainty of evidence in evidence syntheses published in high impact factor nutrition journals: A methodological survey. J. Clin. Epidemiol..

[30] Chan A.W., Tetzlaff J.M., Altman D.G., Laupacis A., Gøtzsche P.C., Krleža-Jerić K., Hróbjartsson A., Mann H., Dickersin K., Berlin J.A. (2013). SPIRIT 2013 Statement: Defining standard protocol items for clinical trials. Ann. Intern. Med..

[31] Moher D., Hopewell S., Schulz K.F., Montori V., Gøtzsche P.C., Devereaux P.J., Elbourne D., Egger M., Altman D.G. (2010). CONSORT 2010 explanation and elaboration: Updated guidelines for reporting parallel group randomised trials. BMJ.

[32] Dwan K., Li T., Altman D.G., Elbourne D. (2019). CONSORT 2010 statement: Extension to randomized crossover trials. BMJ.

[33] Tunn R., Boutron I., Chan A.W., Collins G.S., Hróbjartsson A., Moher D., Schulz K.F., de Beyer J.A., Nejstgaard C.H., Østengaard L. (2024). Methods used to develop the SPIRIT 2024 and CONSORT 2024 Statements. J. Clin. Epidemiol..

[34] Kamioka H. (2023). Current status and issues on the Foods with Function Claims system in Japan: Evidence of functionality of the foods. J. Pharm. Soc. Jpn..

[35] Minozzi S., Gonzalez-Lorenzo M., Cinquini M., Berardinelli D., Cagnazzo C., Ciardullo S., Nardi P.D., Gammon M., Iovino P., Lando A. (2022). The University of Milan Post Graduate Course on Systematic Review Working Group: Adherence of systematic reviews to Cochrane RoB2 guidance was frequently poor: A meta epidemiological study. J. Clin. Epidemiol..

[36] Crocker T.F., Lam N., Jord M., Brundle C., Prescott M., Forster A., Ensor J., Gladman J., Clegg A. (2023). Risk-of-bias assessment using Cochrane’s revised tool for randomized trials (RoB 2) was useful but challenging and resource-intensive: Observations from a systematic review. J Clin. Epidemiol..

[37] Minozzi S., Dwan K., Borrelli F., Filippini G. (2022). Reliability of the revised Cochrane risk-of-bias tool for randomised trials (RoB2) improved with the use of implementation instruction. J. Clin. Epidemiol..

[38] Tomlinson E., Cooper C., Davenport C., Rutjes A.W.S., Leeflang M., Mallett S., Phiting W. (2024). Common challenges and suggestions for risk of bias tool development: A systematic review of methodological studies. J. Clin. Epidemiol..

